# Time-dependent effects in consecutive cycles of prone positioning for acute respiratory failure: insights from the PROVENT-C19 Registry

**DOI:** 10.1186/s44158-025-00318-y

**Published:** 2026-01-03

**Authors:** Nicolò Sella, Annalisa Boscolo, Andrea Cortegiani, Giacomo Bellani, Giuseppe Foti, Silvia De Rosa, Annalisa Pitino, Giovanni Luigi Tripepi, Lucia Cattin, Alessandro De Cassai, Muhammed Elhadi, Giorgio Fullin, Eugenio Garofalo, Leonardo Gottin, Alberto Grassetto, Salvatore Maurizio Maggiore, Elena Momesso, Mario Peta, Tommaso Pettenuzzo, Daniele Poole, Roberto Rona, Andrea Zanoletti, Emanuele Rezoagli, Paolo Navalesi

**Affiliations:** 1 Department of Medicine (DIMED), University of Padova, Padova, PD Italy; 2 Institute of Anesthesia and Intensive Care, Padova University Hospital, Padova, Italy; 3 Department of Precision Medicine in Medical, Surgical and Critical Care (Me.Pre.C.C.), University of Palermo, Palermo, Italy; 4 Department of Anesthesia, Intensive Care and Emergency, Policlinico Paolo Giaccone, Palermo, Italy; 5 Centre for Medical Sciences—CISMed, University of Trento, Trento, Italy; 6 Department of Medicine and Surgery, University of Milano-Bicocca, San Gerardo Hospital, Monza, Italy; 7 CNR-IFC, Institute of Clinical Physiology of Roma, Roma, Italy; 8 CNR-IFC, Institute of Clinical Physiology of Reggio Calabria, Reggio Calabria, Italy; 9 UOC Anestesia e Rianimazione, AULSS8 Berica, Ospedale San Bortolo, Vicenza, Italy; 10 Faculty of Medicine, University of Tripoli, Tripoli, Libya; 11 Anesthesia and Intensive Care, Ospedale all’Angelo, Mestre, Italy; 12 Anaesthesia and Intensive Care, Department of Medical and Surgical Sciences, Magna Græcia University, Catanzaro, Italy; 13 UOC di Anestesia e Terapia Intensiva Cardio-Toraco-Vascolare, Dipartimento di Emergenza e Terapie Intensive, Azienda Ospedaliera Universitaria Integrata di Verona, Verona, Italy; 14 UOC Anestesia e Rianimazione, Ospedale di Vittorio Veneto, Vittorio Veneto (TV), Italy; 15 University Department of Innovative Technologies in Medicine and Dentistry, Gabriele d’Annunzio University of Chieti-Pescara, Chieti, Italy; 16 Department of Anesthesiology, Critical Care Medicine and Emergency, SS. Annunziata Hospital, Chieti, Italy; 17 Anaesthesia and Intensive Care Unit, Ospedali di San Donà di Piave e Jesolo, San Donà di Piave, Italy; 18 Department of Anesthesia and Intensive Care, Santa Maria dei Battuti-Ca’ Foncello Hospital, Treviso, Italy; 19 Anesthesia and Critical Care Unit, Ospedale di Belluno, Belluno, Italy; 20 Anesthesia and Intensive Care Unit, Manerbio Hospital, Manerbio, Italy

**Keywords:** Prone position, Mechanical ventilation, Acute respiratory failure, Arterial oxygenation, Ventilatory ratio, Driving pressure

## Abstract

**Background:**

Prone positioning is recommended for patients with acute respiratory distress syndrome not only to improve oxygenation, but also to reduce lung stress, and lower mortality. The association between improved oxygenation during prone position and reduced mortality is still controversial. In previous studies, oxygenation improvement during the first prone positioning cycle was linked to lower intensive care unit (ICU) mortality, especially with prolonged duration. However, physiological data during subsequent cycles were lacking. This study aims to explore the association between ICU mortality and physiological responses to prone positioning—such as arterial oxygenation, dead space, and respiratory mechanics—and to assess how the cumulative time spent in prone or supine positions across all studied cycles influences outcomes.

**Methods:**

International registry including adult patients who underwent prone positioning for acute hypoxemic respiratory failure due to COVID-19. We measured the difference for arterial partial pressure of oxygen to inspired fraction of oxygen ratio (PaO2/FiO2) and ventilatory ratio between baseline supine position and at either the end of cycle of prone position (Delta-PP) or re-supination (Delta-PostPP), focusing on the cycles following the first one.

**Results:**

We included 1523 patients from 53 centers. Both Delta-PP and Delta-PostPP for PaO2/FiO2 were significantly higher in ICU survivors than in ICU non-survivors for all the analyzed prone positioning cycles (*p* ≤ 0.001 for all comparisons). Delta-PP and Delta-PostPP for ventilatory ratio were significantly lower in ICU survivors than in ICU non-survivors for all the analyzed prone positioning cycles (*p* < 0.05 for all comparisons). No difference in the overall time spent in prone position was found between ICU survivors and non-survivors [61 (38, 84) h vs 58 (32, 85) h, respectively, *p* = 0.175]. The cumulative length of prone position was associated with ICU mortality only for the second prone positioning cycle [OR (95% CI) 0.986 (0.978, 0.994)]. No significant association was observed between the time spent in supine position and ICU mortality for all the analyzed prone positioning cycles.

**Conclusions:**

ICU survivors consistently demonstrated better oxygenation and more stable ventilatory ratio across studied prone positioning cycles, whereas non-survivors showed worsening oxygenation when returning supine and increased ventilatory ratio. Additionally, extending the duration of prone position beyond the second cycle may not significantly impact mortality.

**Supplementary Information:**

The online version contains supplementary material available at 10.1186/s44158-025-00318-y.

## Background

Prone position is recommended in patients with severe or moderate-to-severe acute respiratory distress syndrome (ARDS) receiving invasive mechanical ventilation, as it proved not only to increase arterial oxygenation by recruiting the dorsal area of the lungs and improving ventilation/perfusion matching, but also to attenuate lung stress and strain, and to reduce mortality [1–5].

While before the coronavirus disease 2019 (COVID-19) pandemic prone position struggled to be consistently used [6], after 2020 it has gained wide popularity, so that it has been applied in awake patients receiving non-invasive respiratory support [7] and it has been proposed with renewed interest and enthusiasm also in different clinical scenarios, such as in patients undergoing extracorporeal membrane oxygenation (ECMO) [8] and in those suffering primary graft dysfunction after lung transplantation [9].

During the COVID-19 pandemic, the Prone Positioning for Invasively Ventilated Patients with COVID-19 (PROVENT-C19) Registry, a multicenter international registry, was set up [10]. In this international study, exploring a large cohort of mechanically ventilated COVID-19 patients with acute respiratory failure, the oxygenation improvement, observed either at the end of the first cycle of prone position or after re-supination, was inversely associated with intensive care unit (ICU) mortality, with the lowest death rate in patients experiencing oxygenation improvement both during prone position and after re-supination. Moreover, prolonging the duration of the first cycle of prone position was associated with improved oxygenation and survival, with the longer the duration of the first prone positioning cycle, the smaller the ICU mortality [11].

However, these results were limited to the first cycle of prone position, as no data were analyzed about further cycles. For these reasons, the present study was planned to investigate the associations between ICU mortality and the response to prone position, as assessed by physiological parameters [3] such as arterial oxygenation, and dead space fraction estimates. Secondly, we aimed at ascertaining the association between ICU mortality and the time spent by the patient in either prone or supine position during all the studied cycles.

## Methods

### Study design

The PROVENT-C19 is a multicenter, observational registry originally developed by the Veneto Intensive Care Unit (ICU) Network research group [12], with the endorsement of the Italian Society of Anesthesia, Analgesia, Resuscitation and Intensive Care (SIAARTI), the European Society of Intensive Care Medicine (ESICM), and the European Society of Anaesthesiology and Intensive Care (ESAIC) [10]. There was no funding source for this study.

The registry was designed following the Declaration of Helsinki and the study protocol was firstly approved by the Ethics Committee of the Saint Bortolo Hospital, Vicenza, Italy (Study ID Numbers: 22/21). Data were collected through the workflow methodology and software solution of research electronic data capture (REDCap). Each patient was identified through a patient identification number and all personal information was processed in compliance with the European Union General Data Protection Regulation. Patient consent was obtained according to the national regulations of each participating Institution. In cases the patient was incompetent because of critical illness or the use of sedative or anesthetic drugs, consent could be delayed, and a provision for delayed consent was applied: as soon as competent, each patient was fully informed on what had been done, and a written permission of using data collected was obtained [13]. The patients or their legal surrogates were informed of their right to request that the study procedures be discontinued and their right to refuse the study-related use of their medical records [13]. The Strengthening the Reporting of Observational studies in Epidemiology (STROBE) reporting guideline checklist for observational studies was used for reporting this study (Supplementary Table S1).

### Patients

The PROVENT-C19 Registry included, either prospectively or retrospectively, consecutive adult patients who underwent invasive mechanical ventilation due to COVID-19 related acute respiratory failure and were treated with prone positioning from December 31 st 2019 to January 1 st 2023. COVID-19 infection was laboratory confirmed at polymerase chain reaction test on upper or lower airways samples. Patients were excluded if they refused to provide the consent to participate or if they presented contraindications to prone position, i.e., unstable spinal, pelvic or long bone fractures, severe hemodynamic instability (i.e., mean arterial pressure < 65 mmHg despite vasoactive and inotropic drugs), open abdominal wounds, and late-term pregnancy [3–5]. Also, patients who underwent a single cycle of prone position were not included in the present analysis.

### Data collection

**Fig. 1:**
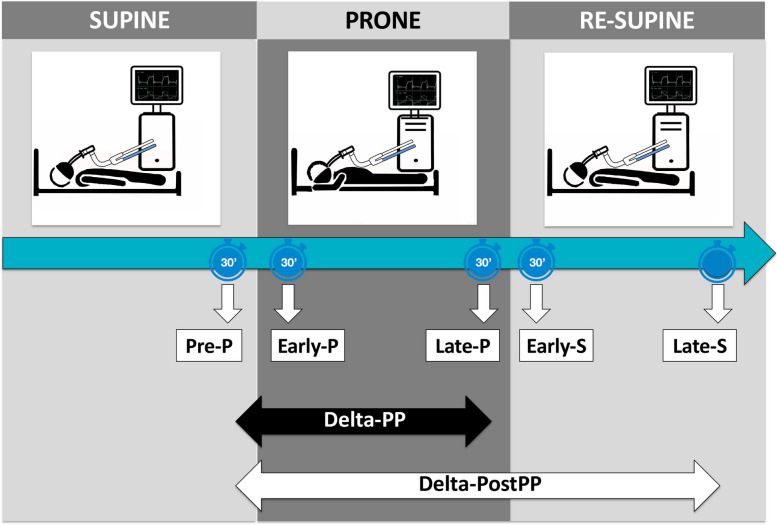
Study design and timeline. These time windows were desirable but not obligatory. The real timing of variable collection was recorded and variables registered more than 30 min later than required by the study design were excluded

The following data were collected and stored for analysis: (i) day and hour of both hospital and ICU admission; (ii) demographic data; (iii) comorbidities; (iv) respiratory support before tracheal intubation; (v) clinical and laboratory parameters (i.e., Sequential Organ Failure Assessment Score—SOFA, Glasgow Coma Score, serum C-reactive protein, serum procalcitonin, serum D-Dimer concentration) at ICU admission; (vi) arterial blood gases at ICU admission and before intubation; *vii)* total number and length of prone positioning cycles with invasive mechanical ventilation during ICU stay. In particular, for each cycle in prone position, ventilator settings (i.e., tidal volume [Vt] normalized by the predicted body weight, respiratory rate, positive end-expiratory pressure [PEEP], fraction of inspired oxygen [FiO_2_]), respiratory mechanics parameters (i.e., driving pressure [DP] [14], static compliance of the respiratory system [Crs]) [14], ventilatory ratio [15], and arterial blood gases values were recorded at different time points as previously described [11] (Fig. 1):

1. pre-prone positioning (pre-P), i.e., within 30 min before the patient was turned prone;
2. early prone positioning (early-P), i.e., within 30 min after the patient being turned prone;
3. late prone positioning (late-P), i.e., within 30 min before the patient was turned supine at the end of the prone positioning cycle;
4. early re-supine positioning (early-S), i.e., within 30 min after the patient being turned supine at the end of the prone positioning cycle;
5. late re-supine positioning (late-S), i.e., within 30 min before the patient was turned prone again for a new prone positioning cycle or before deciding not to proceed to further prone positioning cycles.

Physiological response to prone position was evaluated at different time points (Fig. 1), and values of arterial partial pressure of oxygen to fraction of inspired oxygen ratio (PaO2/FiO2), ventilatory ratio (as proxy for dead space) [16], DP, and Crs were used to calculate:

- Difference in prone position (Delta-PP): the difference between the last available values within the last 30 min in prone and supine position (prior to being turned prone), respectively; i.e., the difference between late-P and pre-P;
- Difference after prone positioning (Delta-PostPP): the difference between the last available value in supine position after the end of the prone positioning cycle and the last available value in supine position before being turned prone (in the last 30 min prior to being turned prone); i.e., the difference between late-S and pre-P.

Since the durations of prone positioning cycles and the intervals of supine positioning between consecutive prone cycles were not standardized, the data collection protocol mandated recording the start and end times of each prone positioning cycle in day and time format, therefore enabling precise calculation of the total time patients spent in supine and prone positions for each studied cycle. Subsequently, the cumulative supination and pronation lapses were calculated from the observed times for each cycle.

### Outcomes

The primary outcome was ICU mortality, thus patients were categorized accordingly in survivors versus non survivors. Secondary outcomes were (i) ICU length of stay, (ii) hospital survival and length of stay; (iii) need for tracheostomy, continuous renal replacement therapy (CRRT), ECMO or extracorporeal carbon dioxide removal (ECCO2R). Also, incidence and outcome of extubation (extubation failure was defined as any episodes of respiratory failure requiring invasive ventilatory support, occurring within seven days after extubation), 28-day ventilator free days, and complications related to prone positioning (i.e., pressure ulcers, endotracheal tube obstruction or accidental removal, vascular lines or chest tube dislodgement, severe hemodynamic instability, acute hemorrhage) [3] were registered.

### Data management

We checked for quality and integrity of the reported data. Missing, extreme or implausible values [17] were returned to the local principal investigator for review and correction. The remaining missing data and data from protocol deviation (e.g., variables registered more than 30 min later than required by the study design) were omitted from the analyses. Complete case analysis was performed.

### Statistical analysis

Categorical data are presented as absolute numbers (n) and percentages (%). Continuous data are given as medians and interquartile ranges.

Data on the first five consecutive cycles of prone position (i.e., from the first up to the fifth one, without any weaning attempts from mechanical ventilation scheduled or performed between them) is analyzed. Further cycles following the fifth one are not considered due to limited sample size and the possible introduction of selection bias.

Between group comparisons for categorical data are performed via Pearson’s chi-squared test or Fisher’s exact test, whereas continuous variables are compared by Wilcoxon’s test. Within group comparisons are performed by Friedman’s test for related pair samples.

The association between cumulative supination or pronation time and mortality is evaluated. For each pronation cycle, univariable logistic regression models are performed for both supine and prone position cumulative times. For each cycle, the effect modification by baseline values of PaO2/FiO2 or ventilatory ratio on the link between cumulative pronation time and mortality was investigated by introducing the corresponding interaction (multiplicative) terms into the model.

To assess the association between major baseline characteristics with ICU mortality, multivariable logistic models are performed separately for each cycle. The tested baseline variables were selected for their recognized clinical value by the study steering committee (“educated guess”) and included: age, gender, PaO2/FiO2 ventilatory ratio, and SOFA score.

To evaluate the link between PaO2/FiO2 or ventilatory ratio with ICU mortality, multivariable logistic models are performed in each phase (Pre-P, Early-P, Late-P, Early-S, and Late-S) and adjusted by baseline characteristics, current PaO2/FiO2 or ventilatory ratio, and their values in previous phase when not collinear. For all logistic models, area under curve (AUC) and their 95% CI are calculated. Logistic regression results are expressed as odds ratios (OR) with 95% confidence intervals (CI).

Physiological responses to prone position at different time points are evaluated by vital status at ICU discharge (ICU alive and ICU dead).

All statistical tests are 2-tailed, and statistical significance is defined as p value < 0.05. All analyses.

have been conducted using R version 4.4.3 (R foundation for Statistical Computing, Vienna, Austria), SPSS version 29.0.2.0 (IBM corporation).

## Results

**Fig. 2:**
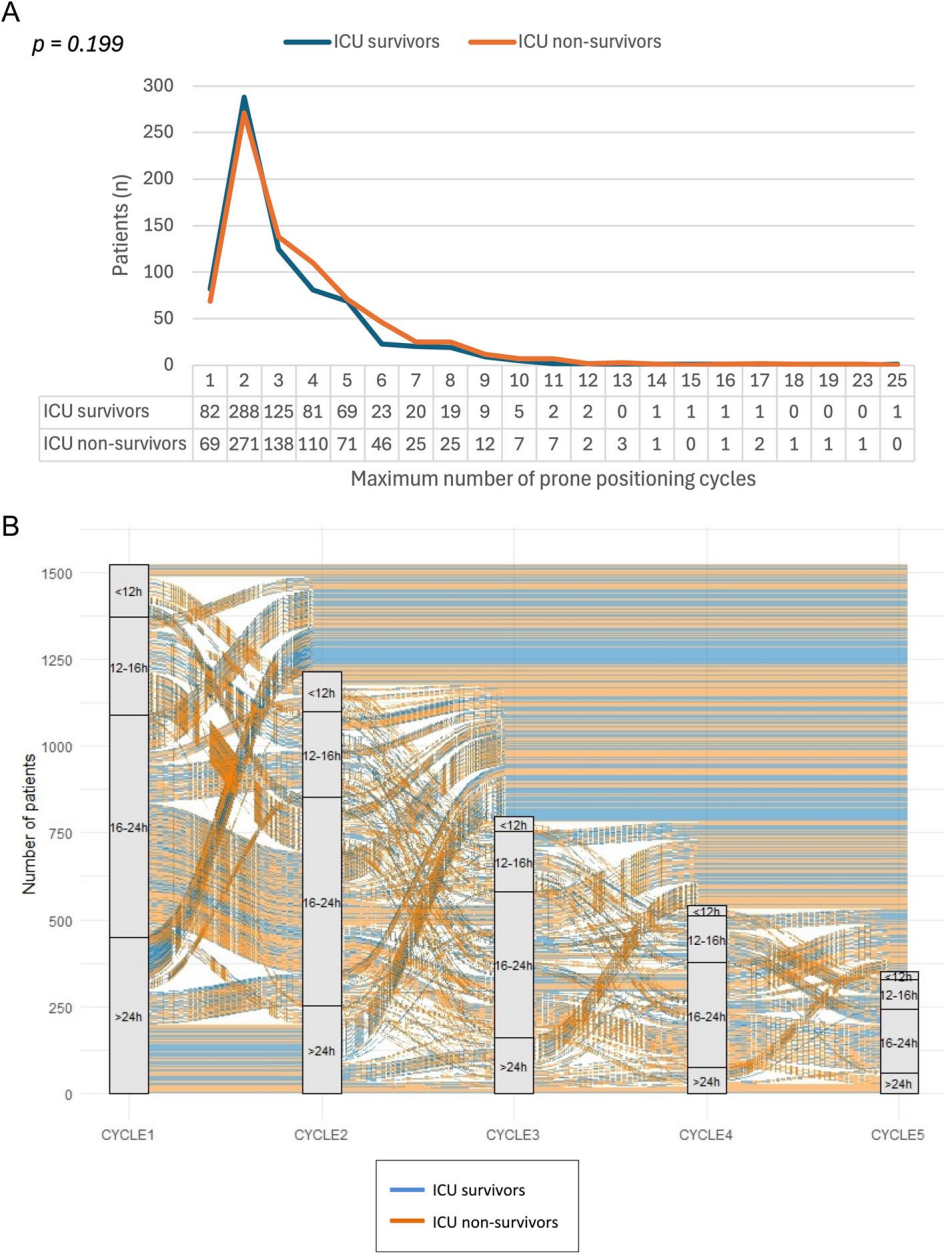
**A** Per-patient distribution of the maximum number of prone positioning cycles. **B** Alluvial plot showing the flow of patient numbers across consecutive prone positioning cycles, grouped by the duration of each cycle (< 6 h, 6–12 h, 12–24 h, > 24 h). The plot tracks how patients transition between the different cycle duration groups over the first five cycles. Patient outcomes of alive or dead upon discharge from the ICU are indicated by the color bands. Abbreviations. ICU, intensive care unit

Among 1816 patients, enrolled from 53 international centers, complete data for the analysis were available for 1523 patients (Supplementary Fig. S1). The baseline characteristics of the study population and the main clinical outcomes are shown in Table 1. Overall, 730 patients (47.9%) were discharged alive from ICU, while 793 patients (52.1%) died during the ICU stay. A total of 818 patients (53.7%) did not survive hospital stay. The per-patient distribution of the maximum number of prone positioning cycles is depicted in Fig. 2, with 1372 patients (90.1%) receiving more than one cycle of prone position and 813 patients (53.4%) turned prone more than twice. No difference in the median number of prone positioning cycles was found between ICU survivors and non-survivors [3 (2, 3) cycles vs 4 (2, 5) cycles, respectively, *p* = 0.199].

**Table 1:** Demographic and baseline characteristics, and main clinical outcomes of the study population

Characteristics	Overall* n* = *1523*	ICU survivors* n* = *730 (47.9%)*	ICU non-survivor* n* = *793 (52.1%)*	*p*-value
Demographics				
Age, *years*	66 (58, 73)	61(53, 69)	69 (62, 75)	** < 0.001**
Male, *n (%)*	1078 (70.8)	502 (68.8)	576 (72.6)	0.097
BMI, *kg/m*^*2*^	28 (26, 33)	29 (26, 33)	28 (25, 32)	0.069
Comorbidities				
COPD, *n(%)*	150 (9.9)	34 (4.7)	116 (14.6)	** < 0.001**
Arterial hypertension, *n (%)*	846 (55.7)	337 (46.3)	509 (64.3)	** < 0,001**
Chronic heart failure, *n (%)*	195 (12.8)	54 (7.4)	141 (17.8)	** < 0.001**
Cerebral vasculopathy, *n (%)*	60 (3.9)	19 (2.6)	41 (5.2)	**0.010**
Diabetes mellitus, *n (%)*	380 (25.0)	146 (20.0)	234 (29.5)	** < 0.001**
Chronic kidney disease, *n (%)*	70 (4.6)	8 (1.1)	62 (7.8)	** < 0.001**
Chronic liver failure, *n (%)*	30 (2.0)	7 (1.0)	23 (2.9)	**0.006**
Cancer, *n (%)*	65 (4.3)	20 (2.7)	45 (5.7)	**0.005**
Immunological deficiency, *n (%)*	115 (7.6)	35 (4.8)	80 (10.1)	** < 0.001**
Before ICU admission				
Corticosteroids before ICU admission, *n(%)*	1069 (70.5)	507 (69.5)	562 (71.4)	0.427
Anticoagulant therapy before ICU admission, *n (%)*	1010 (66.7)	481 (66.0)	529 (67.3)	0.585
Non-invasive respiratory support before ICU, *n (%)*	876 (57.6)	445 (61.0)	431 (54.5)	**0.005**
ICU admission				
IMV at ICU admission, *n (%)*	463 (30.4)	193 (26.4)	270 (34.1)	**0.005**
PaO_2_/FiO_2_ at ICU admission, *mmHg*	87 (67, 117)	90 (70, 120)	84 (65, 114)	** < 0.001**
SOFA at ICU admission	4 (3, 6)	4 (3, 5)	4 (4, 7)	**< 0.001**
White blood cells at ICU admission, × *10*^*9*^*/L*	10 (7, 14)	10 (7, 13)	11 (8, 15)	**0.035**
CRP at ICU admission, *mg/L*	45 (12, 132)	50 (13, 130)	40 (11, 134)	**0.628**
Procalcitonin at ICU admission, *mcg/L*	0.2 (0.1, 0.6)	0.2 (0.1, 0.5)	0.3 (0.1, 0.8)	**< 0.001**
D-Dimer at ICU admission, *mcg/L*	1009 (295, 3166)	951 (366, 2186)	1151 (35, 4167)	**0.372**
Before first prone positioning cycle				
VT, mL/kg PBW	6.8 (6.0, 7.5)	6.7 (6.1, 7.5)	6.8 (6.1, 7.6)	0.437
Respiratory rate, bpm	20 (18, 25)	20 (17, 25)	20 (18, 24)	0.260
PEEP, cmH2O	10 (8, 12)	10 (8, 12)	10 (8, 12)	0.751
Pplat, cmH2O	23 (20, 26)	23 (19, 26)	23 (21, 26)	0.787
Driving pressure, cmH2O	12 (10, 14)	12 (9, 13)	12 (11, 15)	0.900
Crs, mL/cmH2O	38 (32, 50)	40 (33, 50)	37 (30, 50)	0.709
FiO2	0.9 (0.7, 1.0)	0.9 (0.7, 1.0)	1.0 (0.8, 1.0)	0.083
Length of IMV before prone positioning, *hours*	5 (2, 22)	4 (1, 14)	7 (2, 27)	** < 0.001**
During prone positioning cycles				
Continuous NMB,* n (%)*	978 (64.2)	465 (63.7)	513 (64.7)	0.831
iNO,* n (%)*	90 (5.9)	41 (5.6)	49 (6.2)	0.642
Cycles of prone position,* n*	3 (2, 4)	3 (2, 4)	4 (2, 5)	0.199
Overall time in prone position, *hours*	60 (34, 85)	61 (38, 84)	58 (32, 85)	0.175
Pressure injuries,* n (%)*	435 (28.6)	206 (28.2)	229 (28.9)	0.776
Outcomes				
ICU LOS, *days*	14 (9, 24)	16 (10, 27)	13 (8, 21)	** < 0.001**
Hospital LOS, *days*	25 (16, 39)	35 (22, 51)	19 (12, 28)	** < 0.001**
Tracheostomy, *n (%)*	386 (25.3)	227 (31.1)	159 (20.1)	** < 0.001**
Renal replacement therapy, *n (%)*	30 (4.1)	162 (10.7)	132 (16.8)	** < 0.001**
ECMO or ECCO_2_R, *n (%)*	33 (2.2)	9 (1.2)	24 (3.0)	**0.016**
Length of IMV, *hours*	12 (7, 21)	11 (7, 19)	13 (8, 25)	**0.020**

Data are median (I quartile, III quartile) for continuous variables and absolute numbers (percentages) for categorical variables. Results of the test for differences between ICU survivors and ICU non-survivors are reported as *p-value*

*ICU *intensive care unit, *BMI* body mass index, *COPD* chronic obstructive pulmonary disease, *IMV* invasive mechanical ventilation, *PaO*_*2*_*/FiO*_*2*_ arterial partial pressure of oxygen to inspire fraction of oxygen ratio, *SOFA* sequential organ failure assessment, *CRP* C-reactive protein, *ECMO* extracorporeal membrane oxygenation, *ECCO*_*2*_*R *extracorporeal carbon dioxide removal

The multivariable logistic regression models by baseline characteristics on ICU mortality in each prone positioning cycle are reported in Supplementary Table S2. In all cycles of prone position significant associations with ICU mortality were found for age, SOFA score, and values of PaO2/FiO2 and ventilatory ratio measured before the first cycle of prone position, while gender was never associated with the outcome.

### Physiological response to prone position

**Fig. 3:**
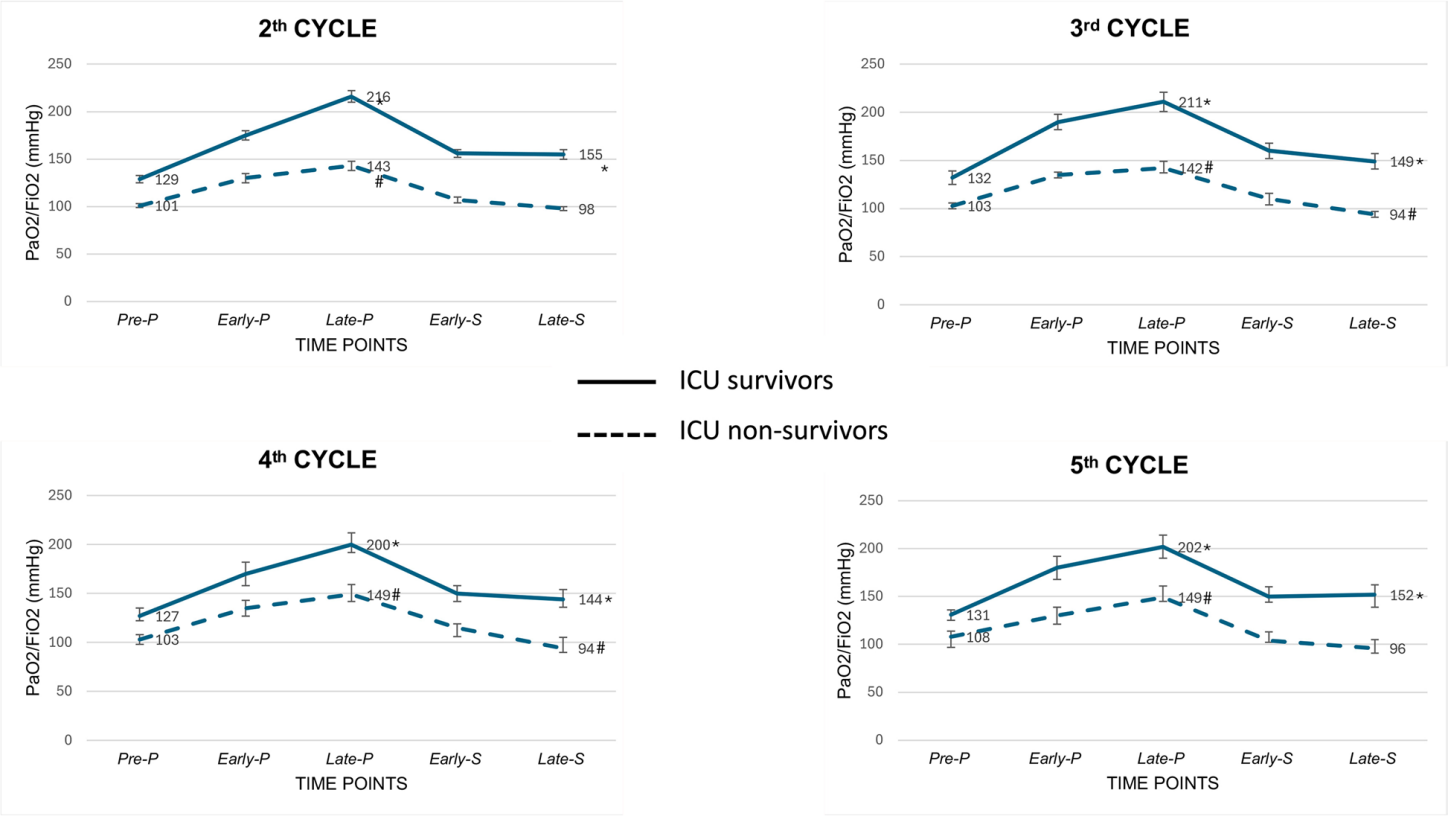
Oxygenation response to consecutive cycles of prone position. Differences between ICU survivors and non-survivors are reported in Table 3. Abbreviations. ICU, intensive care unit. PaO2/FiO2, arterial partial pressure of oxygen to inspired oxygen fraction ratio. * statistically significant difference (p < 0.05) compared to Pre-P value among ICU survivors. # statistically significant difference (p < 0.05) compared to Pre-P value among ICU non-survivors

The response to the consecutive cycles of prone position based on PaO2/FiO2 is depicted in Fig. 3 and in Table 2 and Supplementary Table S3. Among ICU survivors, median PaO2/FiO2 in both late-P and late-S was greater than PaO2/FiO2 in pre-P for all the analyzed cycles. On the contrary, among ICU non-survivors, median PaO2/FiO2, despite being higher in late-P than in pre-P in all the considered cycles, was always not different or even lower in late-S than in pre-P. Both Delta-PP and Delta-PostPP for PaO2/FiO2 were significantly higher in ICU survivors than in ICU non-survivors for all the analyzed prone positioning cycles (*p* ≤ 0.001 for all comparisons). Of note, the temporal trend of PaO2/FiO2 for ICU non-survivors in the analyzed cycles is different compared to trend in the first cycles, when an increase in arterial oxygenation at re-supination was registered also for ICU non-survivors (Supplementary Fig. 2).

**Table 2:** Physiologic response to prone position

Prone position cycle		PaO_2_/FiO_2_ *(mmHg)*			Ventilatory ratio		
		ICU survivors	ICU non-survivors	*p*-value	ICU survivors	ICU non-survivors	*p*-value
2 *(n* = *1523)*	Delta-PP	75 (32, 127)	36 (5, 78)	< 0.001	0.00 (− 0.14, 0.18)	0.07 (− 0.14, 0.31)	** < 0.001**
	Delta-PostPP	21 (− 7, 61)	− 1 (− 24, 21)	< 0.001	0.03 (− 0.2, 0.22)	0.08 (− 0.13, 0.42)	**0.014**
3 *(n* = *780)*	Delta-PP	69 (31, 116)	36 (9, 70)	< 0.001	0.02 (− 0.15, 0.19)	0.09 (− 0.11, 0.33)	**0.006**
	Delta-PostPP	10 (− 15, 42)	− 7 (− 30, 15)	< 0.001	0.02 (− 0.14, 0.21)	0.13 (− 0.10, 0.45)	** < 0.001**
4 *(n* = *522)*	Delta-PP	67 (28, 113)	41 (8, 79)	0.001	0.01 (− 0.2, 0.18)	0.12 (− 0.14, 0.38)	**0.017**
	Delta-PostPP	18 (− 6, 43)	− 8 (− 26, 10)	** < 0.001**	− 0.02 (− 0.29, 0.20)	0.09 (− 0.12, 0.44)	**0.010**
5 *(n* = *339)*	Delta-PP	59 (29, 113)	35 (13, 29)	** < 0.001**	− 0.07 (− 0.26, 0.11)	0.03 (− 0.17, 0.35)	**0.036**
	Delta-PostPP	19 (− 10, 48)	− 5 (− 26, 20)	** < 0.001**	− 0.07 (− 0.25, 0.16)	0.14 (− 0.09, 0.46)	** < 0.001**

Data are median (I quartile, III quartile). Results of the test for differences between ICU survivors and ICU non-survivors are reported as *p-value*

*ICU* intensive care unit, *PaO*_*2*_*/FiO*_*2*_ arterial partial pressure of oxygen to inspire fraction of oxygen ratio

**Table 3:** Cumulative time spent in either prone or supine position, and its association with ICU mortality

	ICU survivors	ICU non-survivors	*p*-value	Odds ratio (95% CI)
Prone position				
*Time spent in prone position, hours*				
● Cycle 2nd *(n* = *1523)* ● Cycle 3rd *(n* = *780)* ● Cycle 4th *(n* = *522)* ● Cycle 5th *(n* = *339)*	19 (16, 30) 35 (31, 46) 52 (47, 62) 68 (63, 77)	18 (15, 23) 36 (32, 43) 54 (49, 62) 70 (65, 81)	**0.033** 0.222 0.166 0.177	**0.986 (0.978, 0.994)** 0.998 (0.992, 1.004) 0.999 (0.994, 1.005) 0.998 (0.992, 1.005)
Supine position				
*Time spent in supine position before being turned prone, hours*				
● Cycle 2nd *(n* = *1523)* ● Cycle 3rd *(n* = *780)* ● Cycle 4th *(n* = *522* ● Cycle 5th *(n* = *339))*	11 (8, 25) 22 (15, 47) 33 (24, 57) 52 (31, 90)	12 (8, 27) 23 (15, 43) 35 (23, 60) 53 (31, 90)	0.328 0.947 0.671 0.798	1.001 (0.997, 1.004) 0.999 (0.997, 1.003) 1.000 (0.998, 1.003) 0.999 (0.997, 1.003)

Data are median (I quartile, III quartile). Results of the Wilcoxon test for differences between the median of ICU survivors and ICU non-survivors are reported as *p-value*. Results of the univariable analyses are reported as odds ratio (OR), 95% confidence interval (CI)

*ICU *intensive care unit, *OR* odds ratio, *95%CI* 95% confidence interval

**Fig. 4:**
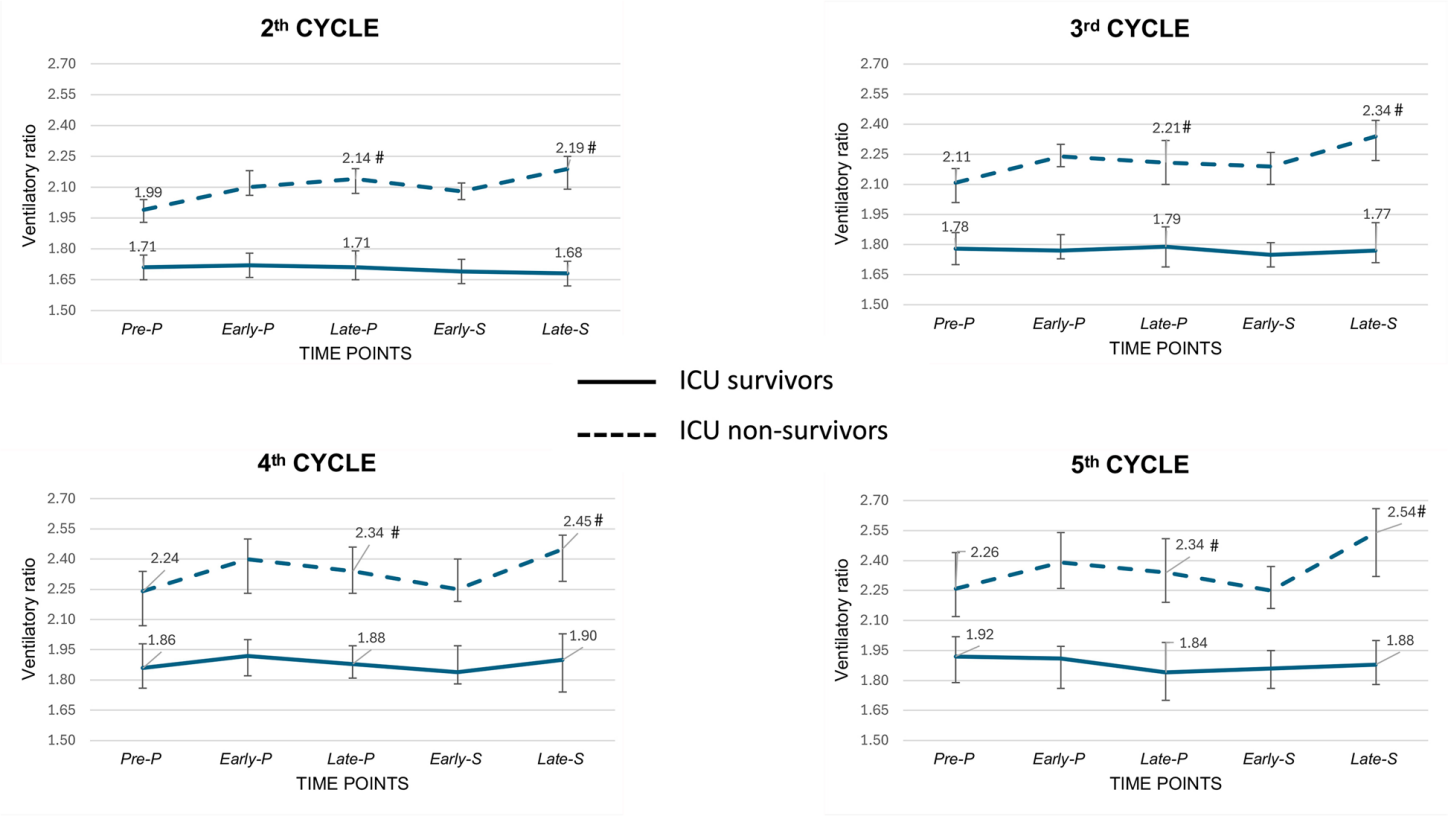
Ventilatory ratio response to consecutive cycles of prone position. Differences between ICU survivors and non-survivors are reported in Table 3. Abbreviations. ICU, intensive care unit. * statistically significant difference (p < 0.05) compared to Pre-P value among ICU survivors. # statistically significant difference (p < 0.05) compared to Pre-P value among ICU non-survivors

The response to the consecutive cycles of prone position with respect to the ventilatory ratio is shown in Fig. 4 and in Table 2 and Supplementary Table S3. While among ICU survivors, median ventilatory ratio was never different in either late-P or late-S as compared to pre-P, among ICU non-survivors median ventilatory ratio increased in both late-P or late-S as compared to pre-P for every cycle of prone position. Delta-PP and Delta-PostPP for ventilatory ratio were significantly lower in ICU survivors than in ICU non-survivors for all the analyzed prone positioning cycles.

The Supplementary Table S4 shows the results of the multivariable logistic regression analysis about the impact on ICU mortality of the PaO2/FiO2 and ventilatory ratio response to the consecutive cycles of prone position, adjusted by measurements on the previous cycles. PaO2/FiO2 values in early-P, late-P, and late-S were independently associated with ICU mortality in every analyzed cycle, while ventilatory ratio values in early-P and late-S resulted to be independent predictors of ICU mortality for all the considered cycles.

Values of DP and Crs at different time-points in consecutives cycles of prone position are illustrated in Supplementary Figs. S3 and S4.

### Time spent in prone and supine position

The time spent in either prone or supine position starting from the second cycle is reported in Table 3 and Fig. 2B. No difference in the overall time spent in prone position was found between ICU survivors and non-survivors [61 (38, 84) h vs 58 (32, 85) h, respectively, *p* = 0.175]. As also depicted in Supplementary Fig. S5, the cumulative length of prone position was associated with ICU mortality only for the second prone positioning cycle [OR (95% CI) 0.986 (0.978, 0.994)]. On the contrary, no significant association was found between the cumulative time spent in supine position and ICU mortality for all the analyzed prone positioning cycles (Supplementary Fig. S6). Of note, baseline values of PaO2/FiO2 or ventilatory ratio did not affect the link between cumulative times and ICU mortality for all the analyzed cycles (p-values for effect modification > 0.05).

## Discussion

In this analysis of a large multicenter international registry, including mechanically ventilated patients undergoing prone position for hypoxemic acute respiratory failure, we found that in consecutive cycles the oxygenation improvement, observed either during prone position or after re-supination, was inversely associated with ICU mortality, with higher PaO2/FiO2 in the ICU survivor group compared to ICU non-survivors. Also, worsening of the ventilatory ratio during prone position and after resupination was associated with a higher ICU mortality.

The different responses in terms of both arterial oxygenation and dead space observed between ICU survivors and non-survivors confirm the results of our previous investigation on the first cycle of prone position [11], which found the increase of oxygenation upon prone position and after re-supination to be associated with ICU mortality. Interestingly, during the prone positioning cycle and after resupination patients discharged alive from ICU presented always PaO2/FiO2 higher than the baseline value before being turned prone; on the contrary, ICU non-survivors, despite improving arterial oxygenation in prone position, did not maintain this beneficial effect after resupination, when the registered PaO2/FiO2 was similar or even lower than the baseline value.

A different trend was observed among ICU non-survivors compared to what was recorded during the first prone positioning cycle, where an improvement in PaO2/FiO2 was seen even in patients who did not survive ICU discharge. These findings, if confirmed by future studies with causal inference, suggest that the arterial oxygenation response might play a role in guiding clinical decisions after the second prone positioning cycle, i.e., no more cycle in case of little or no improvement. Indeed, while no clinical decision should be based solely on the response to the first cycle—as already demonstrated [11]—the trend of arterial oxygenation after the second cycle could be an interesting subject for future research, aiming at optimizing risk stratification and adjusting subsequent treatments accordingly. It’s important to note that, although our results indicate an association between improved arterial oxygenation at the end of prone positioning cycles and mortality, this does not confirm causality. Therefore, we cannot conclude that decisions regarding prone positioning should be made solely based on oxygenation response.

Although several mechanisms may be responsible for improved oxygenation during prone position, including rearrangement of the gas–tissue ratios along the dependent–nondependent regions of the lungs, resolution of dorsal atelectasis, enhanced ventilation/perfusion matching, and a more homogeneous distribution of lung stress and strain [2, 5, 18], the association between improved oxygenation during prone positioning and ICU mortality remains a subject of debate. Indeed, while our findings are in keeping with the work by Camporota et al., who reported that the increase in oxygenation upon prone position was an independent predictor of ICU survival among COVID-19 patients [19], in another study with a different design Albert et al. failed to observe any association between the improvement in arterial oxygenation during prone positioning and survival in 232 ARDS patients [20]. Also, in a physiological study on 13 ARDS patients, Charron et al. demonstrated that improvement in PaO2/FiO2 is not a reliable indicator of the respiratory response during prone positioning, while reduction in alveolar dead space and arterial partial pressure of carbon dioxide are more significant parameters [21].

Our study highlights that, in patients who survived, dead space—estimated using the ventilatory ratio [16]—remained relatively stable during subsequent prone positioning cycles after the first one. In contrast, ICU non-survivors exhibited an increase in ventilatory ratio both during prone positioning and after returning to the supine position.

In ARDS, indices of dead-space ventilation are frequently elevated, particularly in severe cases, due to an increased number of alveolar units exhibiting heterogeneous ventilation/perfusion mismatch [15, 21, 22]. This impairment results from endothelial injury, microvascular plugging with cellular aggregates and thrombi, disordered pulmonary blood flow, and overdistention of alveolar units, which may arise from heterogeneity within the injured lung and/or from the effects of mechanical ventilation itself [22]. Furthermore, in mechanically ventilated patients, the capacity to augment minute ventilation may be compromised or intentionally limited to promote lung protection [15].

These observations of the present study are consistent with prior research on ARDS patients, which showed an association between ventilatory ratio and adverse clinical outcomes [15, 21, 22]. Indeed, impaired ventilation and increased pulmonary dead space, as assessed by the ventilatory ratio, are recognized to be reliable variables that maintain prognostic significance over time [15, 21, 22].

Additionally, our findings reinforce the prognostic value of ventilatory ratio monitoring during prone positioning, supporting previous studies on the first prone positioning cycle, which demonstrated that the patient’s ability to clear carbon dioxide following the first prone positioning was directly associated with survival [11, 23, 24]. Although our results do not establish causation, they emphasize the potential clinical relevance of tracking ventilatory ratio changes throughout and after prone positioning. An increase in physiological dead space—reflected by a rising ventilatory ratio—suggests the possibility of identifying “negative responders” to prone positioning [11, 21, 24], a concept that warrants further validation through dedicated trials designed to assess causality. If confirmed, this perspective could have important clinical implications: (i) an increase in ventilatory ratio following prone positioning may serve as a prognostic marker for higher mortality risk; and (ii) prone positioning should be reconsidered in cases where ventilatory ratio continues to rise. However, further investigations are needed to determine whether dead space elevation linked to lung recruitment maneuvers, such as prone positioning, could serve as a guide for optimizing lung recruitment strategies or explain increased mortality in respiratory failure.

Moreover, the present study shows that after the second cycle cumulative time spent in prone position is not associated with ICU mortality. Since the cornerstone randomized trial by Guerin et al., which proved that turning patients prone reduced both 28-day and 90-day mortality in moderate-to-severe ARDS patients [25], international guidelines strongly recommended to apply 16-h sessions of prone position for patients with severe or moderate-to-severe ARDS [1, 26]. More recently, during the COVID-19 pandemic, prolonged duration of prone position, extended beyond the recommended intervals, resulted to be relatively safe and potentially more effective [27–29]. Indeed, the previously published results of our multicenter study confirmed that the duration of the first prone positioning cycle not only was linearly associated to a higher response in oxygenation either during prone position and after resupination, but it was also independently associated with ICU mortality [11].

While in the first cycle the time spent in prone position is strongly associated with ICU survival [11], the present analysis, focused on subsequent cycles of prone position, shows that such an association fades away during the second cycle and is completely extinguished from the third cycle, so that increasing the duration of time spent prone does not affect the patient's mortality after the second cycle. On the other hand, the cumulative length of supine intervals between consecutive cycles was not associated with ICU mortality, and also the overall number of cycles of prone position did not differ between ICU survivors and non-survivors. These findings are partly in line with the data reported by an ancillary analysis of the ProneCOVID study, which showed in 753 patients with ARDS related to COVID-19 that cumulative duration of prone position of more than 32 h during the first 48 h of ICU admission was not associated with a lower mortality at 60 days compared to a standard prone therapy strategy [30]. On the other hand, Cornejo et al. proved in twenty-four ARDS patients that prone position decreases mechanical determinants of ventilator-induced lung injury, promoting lung recruitment and preventing both cyclic recruitment/derecruitment and tidal hyperinflation [31]. However, the different relationship between the duration of prone positioning and survival across successive cycles, observed in the present study, may be explained by the longitudinal changes of the mechanical properties of the respiratory system during the evolution of acute respiratory failure [32, 33]. Indeed, both in classical ARDS and in COVID-19, respiratory system compliance decreases over time, especially in patients requiring prolonged mechanical ventilation [32, 33], due to the progression of histological lung damage: diffuse alveolar damage, the typical histological characteristic of ARDS, tends to develop preferentially after three days and can progress to fibrosis starting from the second week, potentially resulting in a significant loss of lung compliance and alveolar recruitability [32, 34, 35].

This international, multicenter clinical registry offers several strengths. First, the large sample size enhances the robustness of the results. Second, the inclusion of multiple centers improves the generalizability of the findings. Third, the study benefits from a high level of data granularity, allowing for a more detailed analysis of patient responses to prone positioning.

Nonetheless, some limitations must be acknowledged. As a real-world registry study, it is subject to the inherent constraints of this research design. Notably, potential confounding variables and sources of bias—such as variations in clinical management protocols (e.g., the use of awake prone positioning) and inconsistencies in data collection across different healthcare facilities—were not fully accounted for. Moreover, since the registry contains both prospective and retrospective data, the possibility of information bias due to the retrospective component cannot be excluded.

Another limitation is the study’s exclusive focus on COVID-19 patients, which raises questions about the applicability of these findings to other patient populations. However, by enabling the collection of extensive data from a relatively homogeneous group of patients experiencing acute hypoxemic respiratory failure due to the same underlying disease, COVID-19 provided a valuable model for studying viral pneumonia-related respiratory failure, despite some unique characteristics of the disease.

Also, differences in hospital resources and patient surges during the COVID-19 pandemic may have influenced outcomes. For instance, variations in the duration of the first prone positioning cycle could have been influenced by disparities in ICU staffing and workload. In overwhelmed or understaffed ICUs, healthcare providers may have been unable to sustain prolonged prone positioning or respond promptly to complications, potentially impacting patient survival. Whether these factors played a role in the number and duration of prone positioning cycles and their effect on ICU mortality remains unclear.

Due to the study’s design, prone positioning cycles were analyzed as independent statistical events. However, from a clinical perspective, we cannot exclude the possibility that the response in a previous cycle may have influenced the clinical management of subsequent cycles. This may be true also for the first pronation cycle, which was not considered in the present study. Indeed, the effect of the time spent in the prone position during the first cycle on patient outcomes is likely not negligible, as demonstrated in the previously published paper derived from the same dataset [11].

Lastly, we did not explore the underlying physiological mechanisms driving the clinical response to prone positioning. These limitations highlight the need for cautious interpretation of our results and underscore the importance of further research to deepen our understanding of this topic.

## Conclusions

In our study on adult patients requiring prone positioning for acute hypoxemic respiratory failure due to COVID-19, ICU survivors consistently demonstrated better oxygenation and more stable ventilatory ratio across studied prone positioning cycles, whereas non-survivors showed worsening oxygenation and increased ventilatory ratio when returning supine. Additionally, our results suggest that extending the duration of prone position beyond the second cycle does not significantly impact mortality. These findings highlight the need for individualized approaches to prone positioning strategies. Further research should explore whether optimizing prone position intervals and ventilatory ratio assessment can refine lung-protective strategies in critically ill patients.

## Supplementary Information

Supplementary Material 1. Supplementary Figure S1. Study flowchart. Abbreviations: ICU, intensive care unit.

Supplementary Material 2. Supplementary Figure S2. Oxygenation response (left) and ventilatory ratio response (right) to the first cycle of prone position. Abbreviations. ICU, intensive care unit. PaO2/FiO2, arterial partial pressure of oxygen to inspired oxygen fraction ratio. * statistically significant difference (p < 0.05) compared to Pre-P value among ICU survivors. # statistically significant difference (p < 0.05) compared to Pre-P value among ICU non-survivors.

Supplementary Material 3. Supplementary Figure S3. Driving pressure response to consecutive cycles of prone position. Abbreviations. ICU, intensive care unit.

Supplementary Material 4. Supplementary Figure S4. Static compliance of the respiratory system response to consecutive cycles of prone position. Abbreviations. Crs, static compliance of the respiratory system. ICU, intensive care unit.

Supplementary Material 5. Supplementary Figure S5. Cumulative time spent in prone position. Abbreviations. ICU, intensive care unit.

Supplementary Material 6. Supplementary Figure S6. Cumulative time spent in supine position. Abbreviations. ICU, intensive care unit.

Supplementary Material 7.

## Data Availability

Individual participant data that underlie the results reported in this article, after de-identification, data dictionary, study protocol, statistical analysis plan, informed consent form, and analytic code will be available to any researchers who provide a methodologically sound proposal, immediately following publication and without end date. Proposals should be directed to the corresponding author. To gain access, data requestors will need to sign a data access agreement.

## Abbreviations

ARDS: Acute respiratory distress syndrome
COVID-19: Coronavirus disease 2019
CRRT: Continuous renal replacement therapy
Crs: Static compliance of the respiratory system
Delta-PP: Difference in prone position
Delta-PostPP: Difference after prone positioning
DP: Driving pressure
early-P: Early prone positioning
early-S: Early re-supine positioning
ECCO2R: Extracorporeal carbon dioxide removal
ECMO: Extracorporeal membrane oxygenation
FiO2: Fraction of inspired oxygen
ICU: Intensive care unit
late-P: Late prone positioning
late-S: Late re-supine positioning
PaO2/FiO2: Arterial partial pressure of oxygen to fraction of inspired oxygen ratio
PEEP: Positive end-expiratory pressure
pre-P: Pre-prone positioning
SOFA: Sequential organ failure assessment score
Vt: Tidal volume
