# Supplementary figures and images for: Time-dependent effects in consecutive cycles of prone positioning for acute respiratory failure: insights from the PROVENT-C19 Registry
Source: J Anesth Analg Crit Care. 2026 Jan 3;6:15. doi: 10.1186/s44158-025-00318-y (PMC12866320; doi:10.1186/s44158-025-00318-y)

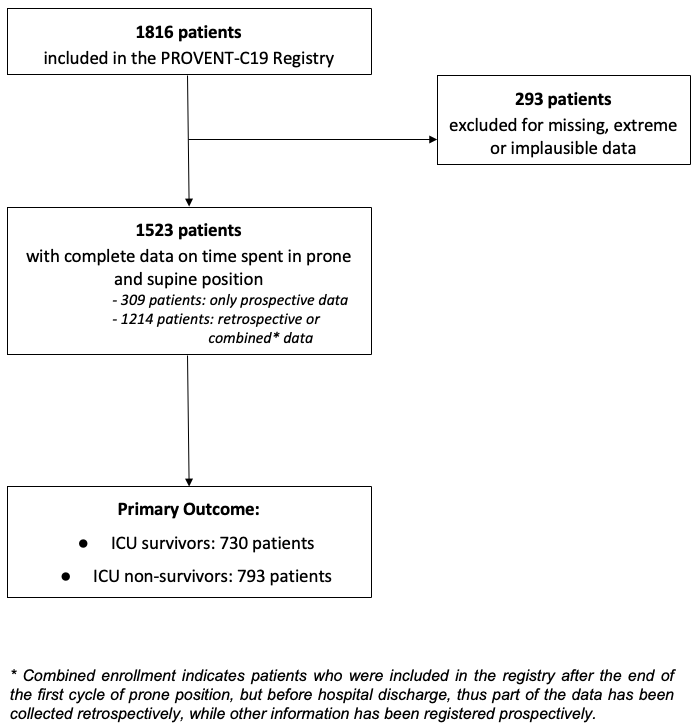

Supplement: Supplementary file 1 — Supplementary Material 1. Supplementary Figure S1. Study flowchart. Abbreviations: ICU, intensive care unit. [file 44158_2025_318_MOESM1_ESM.tiff]

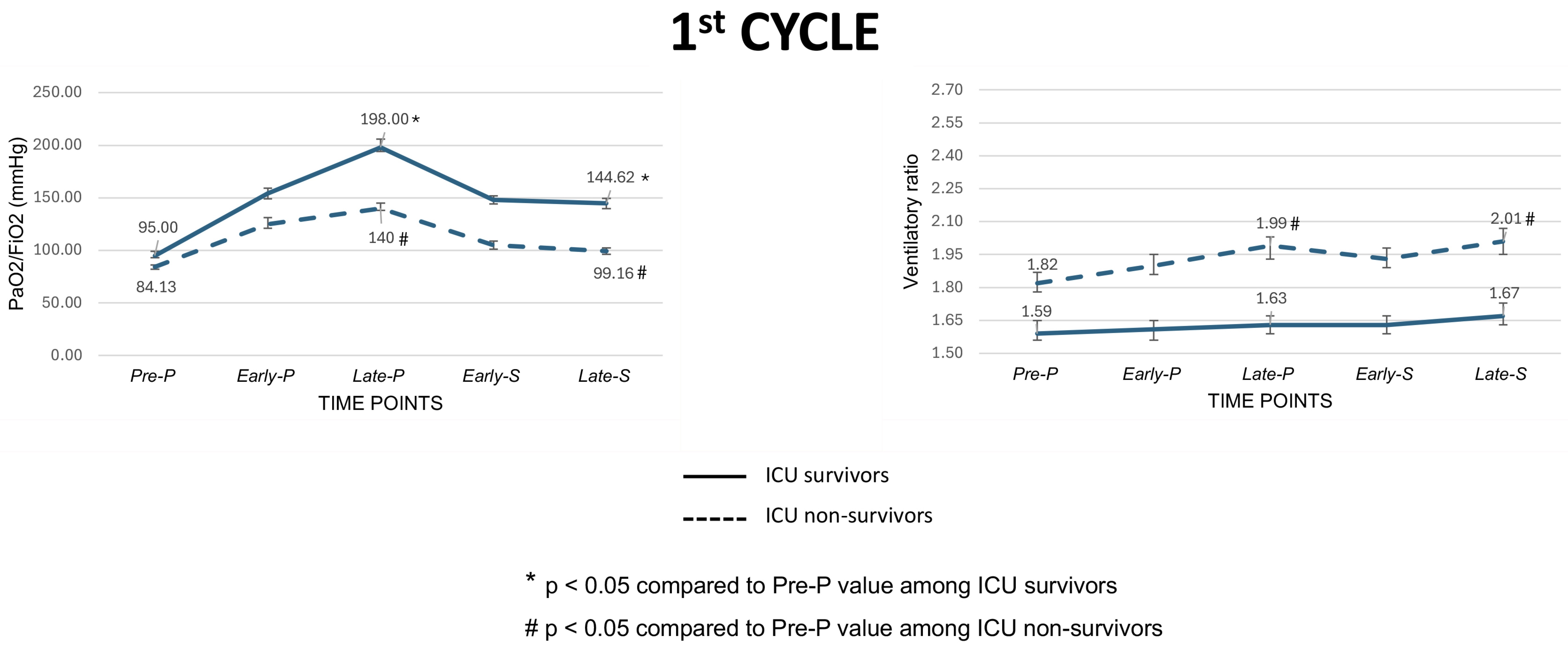

Supplement: Supplementary file 2 — Supplementary Material 2. Supplementary Figure S2. Oxygenation response (left) and ventilatory ratio response (right) to the first cycle of prone position. Abbreviations. ICU, intensive care unit. PaO2/FiO2, arterial partial pressure of oxygen to inspired oxygen fraction ratio. * statistically significant difference (p < 0.05) compared to Pre-P value among ICU survivors. # statistically significant difference (p < 0.05) compared to Pre-P value among ICU non-survivors. [file 44158_2025_318_MOESM2_ESM.jpg]

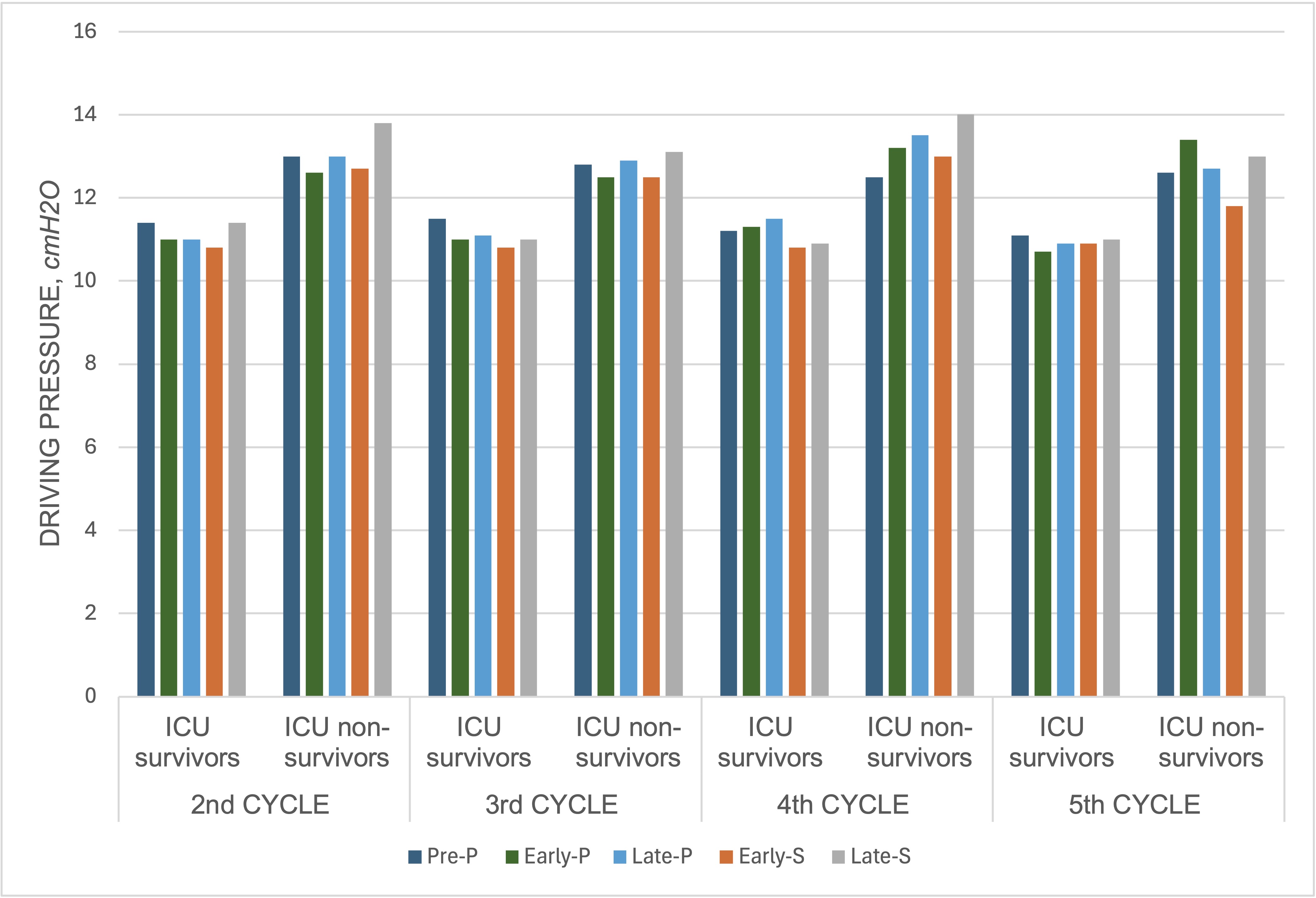

Supplement: Supplementary file 3 — Supplementary Material 3. Supplementary Figure S3. Driving pressure response to consecutive cycles of prone position. Abbreviations. ICU, intensive care unit. [file 44158_2025_318_MOESM3_ESM.jpg]

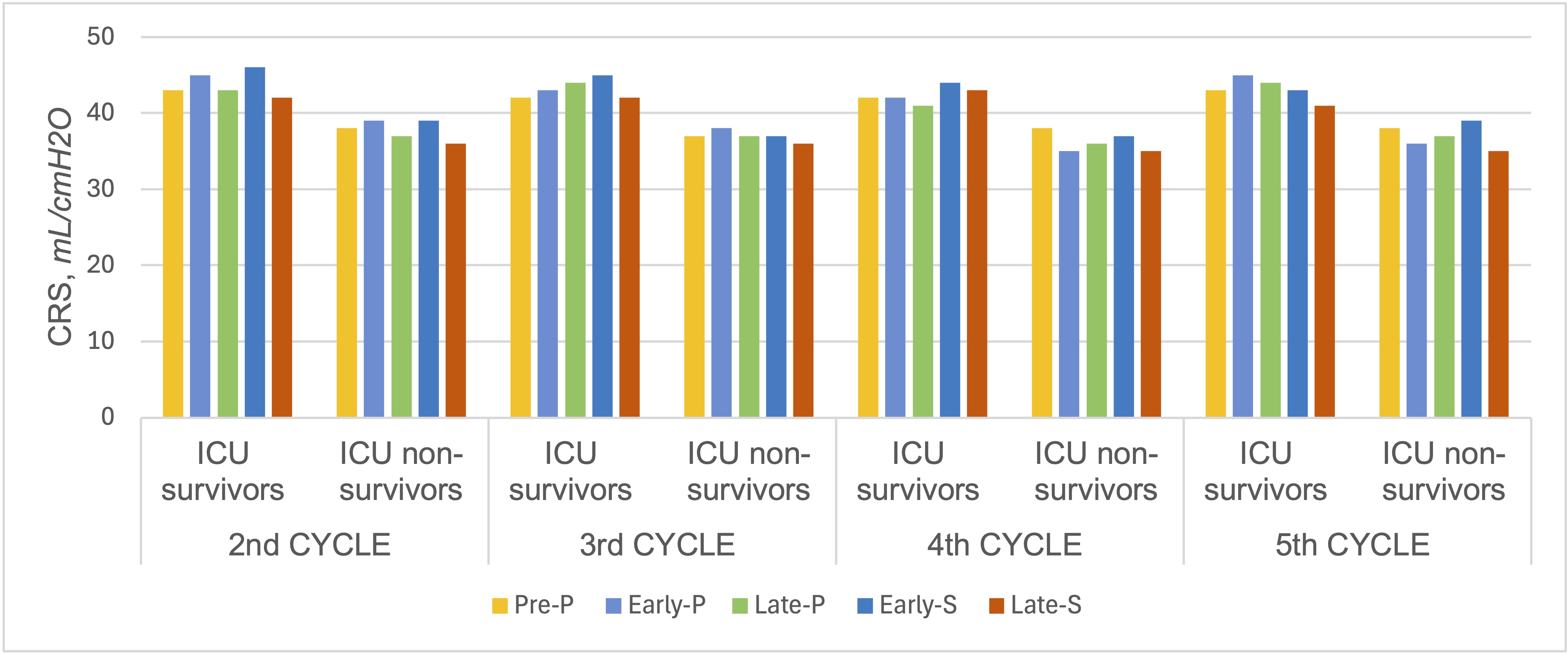

Supplement: Supplementary file 4 — Supplementary Material 4. Supplementary Figure S4. Static compliance of the respiratory system response to consecutive cycles of prone position. Abbreviations. Crs, static compliance of the respiratory system. ICU, intensive care unit. [file 44158_2025_318_MOESM4_ESM.jpg]

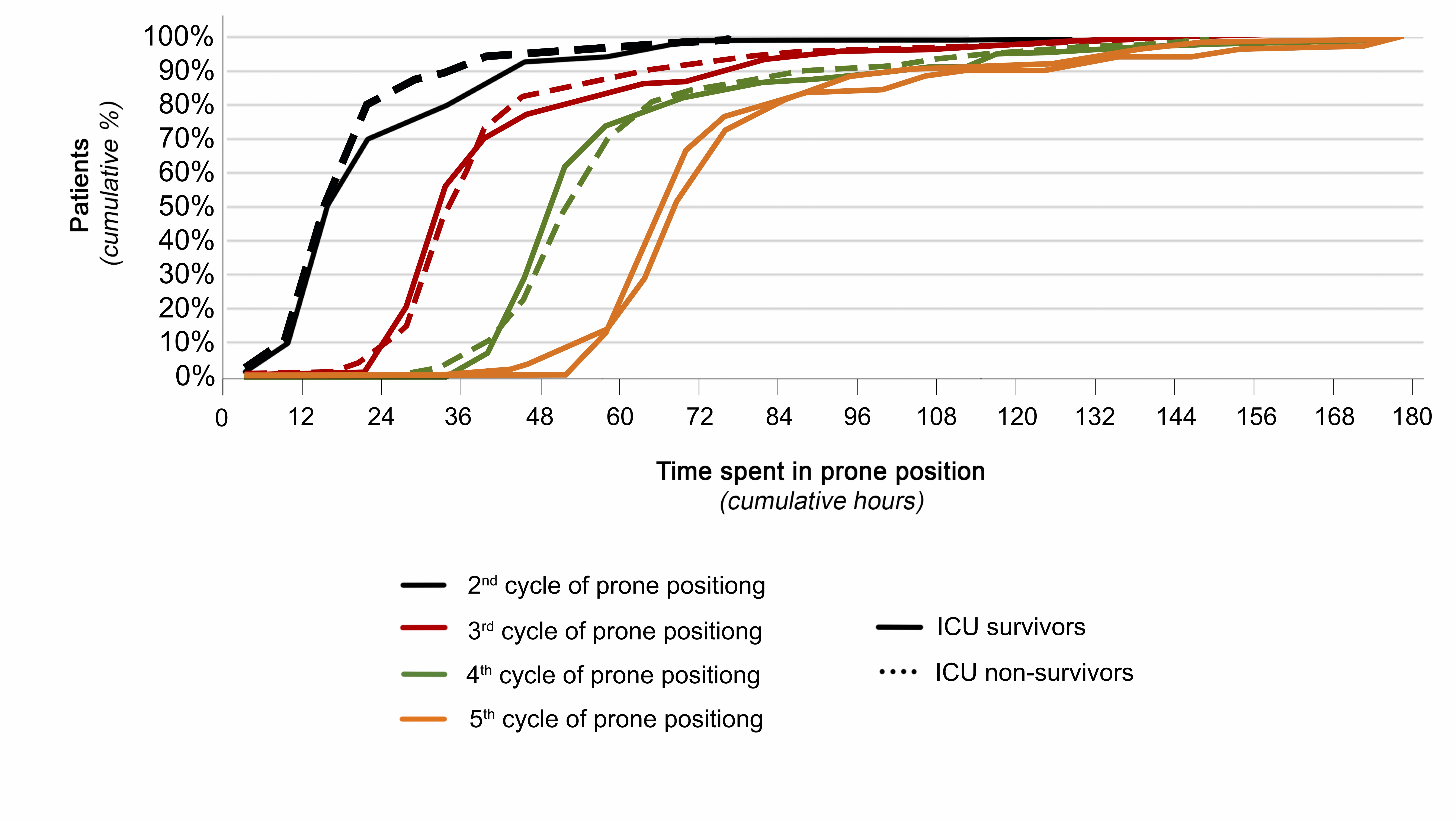

Supplement: Supplementary file 5 — Supplementary Material 5. Supplementary Figure S5. Cumulative time spent in prone position. Abbreviations. ICU, intensive care unit. [file 44158_2025_318_MOESM5_ESM.jpg]

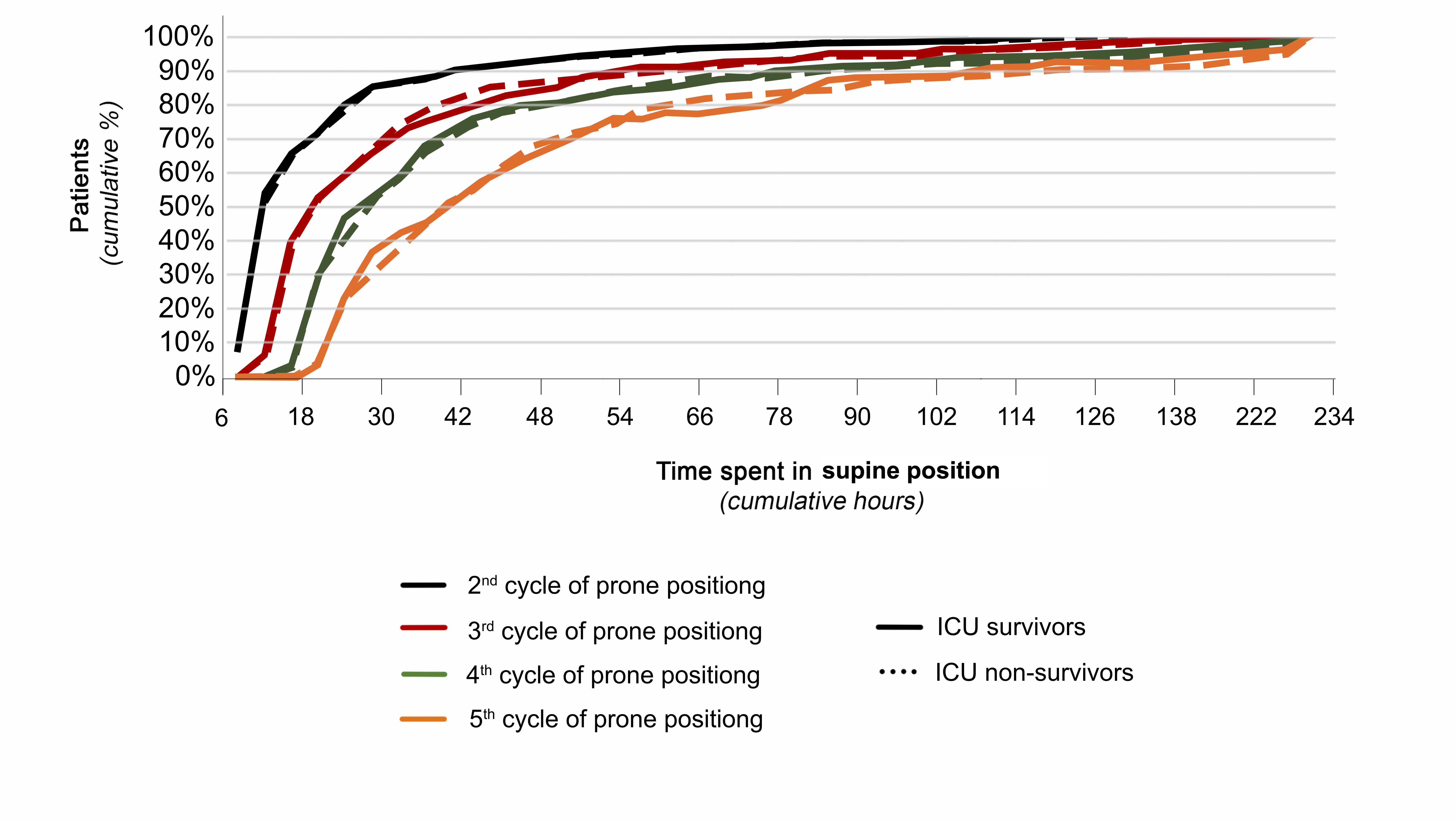

Supplement: Supplementary file 6 — Supplementary Material 6. Supplementary Figure S6. Cumulative time spent in supine position. Abbreviations. ICU, intensive care unit. [file 44158_2025_318_MOESM6_ESM.jpg]
